# The Human Antimicrobial Protein Bactericidal/Permeability-Increasing Protein (BPI) Inhibits the Infectivity of Influenza A Virus

**DOI:** 10.1371/journal.pone.0156929

**Published:** 2016-06-06

**Authors:** Olaf Pinkenburg, Torben Meyer, Norbert Bannert, Steven Norley, Kathrin Bolte, Volker Czudai-Matwich, Susanne Herold, André Gessner, Markus Schnare

**Affiliations:** 1 Institute for Immunology, Philipps-University of Marburg, Marburg, Germany; 2 Department for HIV and other Retroviruses, Robert Koch Institute, Berlin, Germany; 3 Laboratory for Cell Biology, Philipps-University of Marburg, Marburg, Germany; 4 Institute for Virology, Philipps-University of Marburg, Marburg, Germany; 5 Department of Internal Medicine II, University of Giessen Lung Center and German Center for Lung Research, Giessen, Germany; 6 Institute for Clinical Microbiology and Hygiene, University Regensburg, Regensburg, Germany; German Primate Center, GERMANY

## Abstract

In addition to their well-known antibacterial activity some antimicrobial peptides and proteins (AMPs) display also antiviral effects. A 27 aa peptide from the N-terminal part of human bactericidal/permeability-increasing protein (BPI) previously shown to harbour antibacterial activity inhibits the infectivity of multiple Influenza A virus strains (H1N1, H3N2 and H5N1) the causing agent of the Influenza pneumonia. In contrast, the homologous murine BPI-peptide did not show activity against Influenza A virus. In addition human BPI-peptide inhibits the activation of immune cells mediated by Influenza A virus. By changing the human BPI-peptide to the sequence of the mouse homologous peptide the antiviral activity was completely abolished. Furthermore, the human BPI-peptide also inhibited the pathogenicity of the Vesicular Stomatitis Virus but failed to interfere with HIV and measles virus. Electron microscopy indicate that the human BPI-peptide interferes with the virus envelope and at high concentrations was able to destroy the particles completely.

## Introduction

Influenza is a very common infectious disease and the causing agent Influenza A virus is a very successful pathogen. It constantly circulates in many animal hosts, such as humans, pigs, horses, dogs and birds. Annual epidemics of seasonal influenza result in millions of humans worldwide infected. This causes a prominent health and economic risk [1]; influenza pandemics can also have devastating effects globally, resulting in millions of deaths [2].

Influenza A virus (IAV) is an enveloped negative-sense single-stranded RNA-virus of the orthomyxovirus family. Subtypes of IAV expressing different neuraminidase and hemagglutinin proteins are able to infect a variety of hosts. Hemagglutinin thereby interacts with either α-2,3- or α-2,6-sialiated (SA) proteins and enters the cells via endocytosis [3] and therefore determining host tropism. Thereafter, the endosome is acidified which results in the fusion of the virus envelope with endosomal membrane releasing the viral genome into the cytoplasm. Then the viral RNA-protein complex (RNP) translocates to the nucleus where the negative RNA is either replicated into a positive RNA-strand or transcribed to mRNA by the viral encoded RNA-dependent RNA-polymerase. After that the viral mRNAs leave the nucleus and are exported to the cytoplasm for translation. This will most likely result in a total of 11or 12 viral proteins but the precise number of viral proteins is still under debate and might differ in different host cells.

The budding of the progeny virus occurs via the neuraminidase activity of NA. These will destroy the SA moieties of the cellular and the viral glycoproteins and free the active sites of the viral proteins in the envelope to allow for a new infections cycle.

The nonstructural protein NS1 inhibits host interferon-mediated antiviral responses and thus promotes the pathogenesis of IAV [4].

Today we know of 16 HA and 9 NA subtypes of IAV infecting birds. Recently, two additional subtypes of IAV which are bat-derived were identified. These subtypes were termed H17N10 and H18N11, respectively [5,6]. These new findings raising the possibility of bats serving as a reservoir for new subtypes of IAV causing a possible thread of humans. In humans 2 subtypes circulate: H1N1 and H3N2 (H2N2 strains were also circulating in humans from 1957 to 1968). Overall, the HA subtypes are classified into two groups (or lineages) based on their antigenic properties and their major structural features [7–10].

The infectivity of Influenza A virus is restricted by mechanisms of the innate immune response to avoid the binding and/or invasion of the host epithelial cells especially in the lung. One mechanism of the epithelial cells to avoid binding and/or invasion is by the action of antimicrobial proteins and peptides (AMPs).

AMPs are crucial mediators of the innate immune system. In particular AMPs protect the epithelial surfaces of the body and prevent the invasion of pathogens into the host organism.

The potency of AMPs against bacteria is well known and demonstrates that AMPs not only interact with bacterial cell membrane to destroy bacteria [11].

Recently it was shown that some AMPs influence the infectivity of viruses as well. For example the defensin cathelicidin blocks the replication of IAV *in vitro* and therefore the application of cathelicidin protects mice against an infection with IAV in a prophylactic setting [12]. The exact molecular mechanism of the antiviral effect of cathelicidin remains currently unknown. Moreover the human α-Defensin human neutrophil peptide 1 (HNP-1) was shown to display anti-HIV activity. HNP-1 inhibits the binding of the virus to its coreceptor (CCR5 und CXCR4), the endocytosis of the virus into the target cell as well as the release of the HIV-genome from the endosome into the cytoplasm. However HNP-1 did not inhibit the endocytosis of Influenza A virus displaying some selectivity of the AMPs in their tropism [13]. These results clearly demonstrate that defensins not only display antimicrobial activity but in addition are active against viruses as well.

*Bactericidal/permeability-increasing protein* (BPI) belongs to the class of AMPs. In contrast to the above mentioned defensins BPI due to the 55 kDa molecular size of the protein is structurally much more complex than the peptides, which are in the range of 3–5 kDa. The BPI protein family comprises of more than 10 members but only BPI itself displays a strong antimicrobial activity. BPI acts bactericidal against gram negative bacteria, neutralizes bacterial lipopolysaccharide (LPS) from the cell wall of gram negative bacteria and opsonizes bacteria if bound to the bacterial cell wall. On the other hand the closely related protein *lipopolysaccharide binding protein* (LBP) binds LPS with the same molecular moieties in comparison to BPI. This binding however results in the recognition of LPS by the immune system [14]. Recently the *palate*, *lung*, *and nasal epithelium clone associated proteins* (PLUNCs) were added to the BPI protein family. The PLUNC subfamily is not closely related to BPI on the primary amino acid sequence level. However computational predictions of the protein structure of these proteins reveals a surprising similarity with BPI and LBP. The PLUNC proteins are divided into short (S) and long (L) PLUNC proteins. SPLUNCs are homologous to the N-terminal part of BPI whereas LPLUNC proteins are homologous to the complete BPI protein. All PLUNC proteins are expressed in the nasopharyngeal cavity and only SPLUNC1 and LPLUNC1 are found to be expressed in the respiratory system. SPLUNC1 is synthesized by epithelial cells of the upper respiratory tract and LPLUNC1 is associated with goblet cells in the proximity of ciliated epithelial cells of the upper airways. Hence SPLUNC1 and LPLUNC1 are differentially expressed [15].

## Material and Methods

### Ethics Statement

The local ethics committees of Justus-Liebig-University Giessen (Ethikkommission des Fachbereichs Medizin der Justus-Liebig-Universität Giessen) and Philipps-University Marburg (Ethikkommission des Fachbereichs Medizin der Philipps-Universität Marburg) approved the use of human blood samples for this study. The participants provided their written consent to participate in this study. The consent procedure was approved by the ethics committee.

### BPI peptides

Purified BPI was purchased from Athens Research & Technology (Athens, Georgia, USA). The peptides were synthesized by GeneScript (Piscataway, NJ, USA) and are listed in table 1. Except the peptide for the active LL-37 which has an aa sequence as follows: LLGDFFRKSKEKIGKEFKRIVQRIKDFLRNLVPRTES. All peptides had a purity of >95%.

**Table 1:** BPI peptides used.

Name of the peptide	amino acid sequence	charge
huBPI-Peptid:	NANIKISGKWKAQKRFLKMSGNF**D**LSI	+5
mBPI-Peptid:	**D**ASIKINGKWMSRKNFLKAGGNF**E**LSI	+3
Mut.1 (1:N/D):	**D**ANIKISGKWKAQKRFLKMSGNF**D**LSI	+4
Mut.2 (11:K/M):	NANIKISGKWMAQKRFLKMSGNF**D**LSI	+4
Mut.3 (1:N/D;11:K/M):	**D**ANIKISGKWMAQKRFLKMSGNF**D**LSI	+3
Mut.4 (1:D/N;11:M/K):	NASIKINGKWKSRKNFLKAGGNF**E**LSI	+5

Negative charged amino acids are marked in bold and positive charged amino acid are under-lined.

### Stimulation of human neutrophilic granulocytes

After sedimentation of erythrocytes from human buffy coats through 10 mL 6% Dextran T500 in 0.9% NaCl (Roth) for 20 min granulocytes were isolated by density gradient centrifugation using Percoll PLUS (GE). Therefore, 5 mL of 5 x 10^7^ cells/mL were overlaid on Percoll PLUS gradient consisting of two layers. On the bottom 4 mL of 76% (1,099 g/mL) Percoll PLUS solution and 4 mL 61.5% (1,080 g/mL) Percoll PLUS-solution. The Percoll solution were prepared in HBSS. Thereafter the cells were separated on the Percoll-gradient by centrifugation (700 x g, 30 min at room temperature) and the layer between 76% and 61.5% contained the granulocytes. The cells were resuspended in RPMI complete (PAA Laboratories, Pasching, Austria) without antibiotics and used for stimulation experiments. For the analysis of the purity of the isolated granulocytes, fluorescein isothiocyanate-conjugated anti-CD66b monoclonal antibody (mouse IgM, clone G10F5, BD Biosciences, Heidelberg, Germany) and, for macrophages, allophycocyanin-conjugated anti-CD14 monoclonal antibody (mouse IgG1, clone M5E2; BD Biosciences) were used at 5 –l x 10^6^ cells. Nonconjugated mouse IgG1 (clone MOPC-21; BD Biosciences) was used as an isotype control. After Fc-binding sites were blocked to prevent unspecific binding, antibodies were applied for 20 min at 4°C for surface staining. After the cells were washed with PBS-1% fetal calf serum, analysis was performed by flow cytometry. All flow cytometry measurements were performed with a FACSCalibur cytometer using FlowJow and CellQuest software (BD Biosciences).

To induce the degranulation of the granulocytes the cells were cultured in the presence of purified Influenza A virus subtypes (H1N1, H3N2 and H5N1) (0.5, 1, 2, 4, and 8 MOI as determined by plaque test) or left unstimulated. After the indicated time points supernatant were collected and the content of BPI in the supernatant was determined via ELISA.

The concentration of human BPI was determined via a specific ELISA as described elsewhere (Aichele, 2006). Briefly, a rat-anti-mouse IgG monoclonal antibody (Bio-Rad AbD Serotec, Puchheim, Germany) in 0,1 M NaHCO_3_ was coated overnight at 4°C followed by blocking the wells with PBS/10%FCS containing a mouse anti-human BPI antibody (Hycult Biotechnology, mAB HM2041) for 6 h at room temperature. Thereafter, standard (human BPI, Athens Research & Technology) and samples were incubated overnight at 4°C. Finally, the detection of the binding was determined by the incubation of a rabbit anti-human BPI antiserum (Hycult Biotechnology, pAB HP9022) followed by a donkey anti-rabbit peroxidase coupled antiserum (dianova, Hamburg, Germany). Each antibody was incubated for 2 h at room temperature. As substrate TMB super sensitive (BioFX, Sur Modics, Eden Prairie, USA) was used and the reaction was stopped by adding 1 M H_3_PO_4_ (pH 8.2). The OD was measured with an E max Precision Microplate Reader (Molecular Devices, Biberach, Germany) at 450 nm and analyzed with the „Softmax Pro”software.

### Viruses and electron microscopy

Influenza A-Virus strain A/PR/8/34 (H1N1) was purified from the allantois fluid. The strains A/Aichi/2/68 (H3N2) and rg A/Vietnam//1203/04 (H5N1) [16] were purified from cell culture supernatants of infected BHK cells. The IAV strain rg A/Vietnam//1203/04 (H5N1) harbours only hemagglutinin as well as neuraminidase form H5N1 and the rest of the virus is of strain A/PR/8/34 origin. The IAV clinical isolate H5N1 is the previously described virus A/Thailand/1(Kan-1)/2004 strain [17]. Measles virus strain Edmonston was obtained from the cell culture supernatant of infected Vero cells and VSV-GFP was propagated in HeLa cells and purified by dense gradient centrifugation.

For negative staining, purified virus particles were fixed in 1 x PBS containing 6% (v/v) formaldehyde. 5μL particle solution was applied to carbon coated 400 mesh copper grids. Grids were washed twice with distilled water and negatively stained with 2% (w/v) uranyl-acetate for 30 sec. Transmission electron microscopy was carried out using a JEOL TEM 2100 at 120kV. Micrographs were recorded with a fast-scan 2k x 2k CCD camera F214 (TVIPS, Gauting, Germany).

### MDCK infection and detection of the virus

Protease-deficient MDCK(H) cells [18] were infected with 500 PFU/well Influenza A virus strain APR8 (H1N1) or Aichi (H3N2) or Vietnam (H5N1) or Thailand (H5N1) for 1 h. These cells are not able to produce infectious virus particles and therefore a single infection cycle can be detected. Before the infection the virus was incubated in the presence or absence of the indicated amount of human BPI-peptide or control peptides for 1 h. After the infection the supernatant was replaced by growing media and the cells were incubated for additional 13 h except for Thailand (H5N1) which was incubated for 8 h. Thereafter, the multiplied virus could be visualized inside the infected cells by the detection of the viral nucleoprotein. Prior to the staining the cells were fixed with 4% paraformaldehyde and permeabilized with 0.3% of Triton-X-100. After that the fixed and permeabilized cells were incubated with the mouse anti–nucleoprotein IAV monoclonal antibody [19]. The binding of the antibody was detected by a secondary antibody coupled to HRP (donkey anti-mouse IgG-HRP, dianova) and adding of the reagent TMB Super Sensitive One Component HRP Microwell Substrate (BioFX). Colour development was stopped by adding 1 M H_3_PO_4_ and detected with an E max Precision Microplate Reader (Molecular Devices) at 450 nm and analyzed with the „Softmax Pro”software.

### Plaque test for VSV

BHK-21 cells (ATCC HTB-55) were infected with 360 PFU/well of VSV-GFP virus. Prior to infection the VSV was incubated with 20 μg/mL of human BPI and mouse BPI-peptide, respectively or remained untreated for 1 h. After 1.5 h of infection the virus was removed and the cells were overlaid with MEM medium containing penicillin and streptomycin, 1% L-Glutamine, 10% FCS and 1.25% Avicel (Sigma-Aldrich). The cells were incubated for 42 h. Thereafter, the supernatant was removed from the cells and the cells were fixed with 10% of formaldehyde at 4°C for 1 h and stained with 0.1% crystal violet for 1 h at 4°C after washing of the formaldehyde. After extensive washing of the excessive crystal violet the plaques were counted.

### Infection of Calu3 cells with IAV

50–70% confluent Calu-3 cells (ATCC CCL-10) were infected with 300 PFU/well Influenza A virus strain A/Aichi/2/68 (H3N2). Prior to the infection the virus was incubated in the presence or absences of the indicated amount of human or mouse BPI-peptide for 1 h. Thereafter, the virus solution was removed and the cells were incubated for 24, 48, 72, and 96 h. The virus amount in the supernatant was analysed by adding an aliquot of the supernatant to MDCK (H) cells and performing the detection of the virus as outlined above in the part MDCK infection and detection of the virus. Furthermore, also the release of CCL5 into the supernatant of the infected Calu-3 cells was determined via a specific ELISA as recommended by the manufacturer (Peprotech, Hamburg, Germany). The OD was measured with an E max Precision Microplate Reader (Molecular Devices) at 450 nm and analyzed with the „Softmax Pro”software.

### Isolation of PBMCs and stimulation

Human PBMCs were isolated by dense gradient centrifugation from buffy coats. Briefly, cells from the buffy coat were pipetted onto lymphocyte separation medium (Ficoll-solution; 1,077 g/mL, PAA) and centrifuged at 670 x g for 30 minutes at room temperature (20°C). The upper layer contained the PBMCs. The cells were resuspended in RPMI complete substituted with 2% of AB serum (human serum of blood group AB positive). Thereafter, the cells were seeded and stimulated with LPS (100 ng/mL, Sigma-Aldrich), CpG 2216 (2 μM, Biomers, Ulm, Germany), purified influenza A virus (MOI 2) in the presence and absence of human and mouse BPI-peptides, and left unstimulated. After 20 h the supernatant was collected and the release of IFNα was determined by a specific ELISA.

Therefore, anti-human IFNα coating antibody (BenderMedSystems, Wien, Austria) was used to coat a microtiter plate and the binding of recombinant IFNα (Peprotech) as standard or the samples were detected by a secondary anti-human-IFNα HRP-conjugated antibody (BenderMedSystems). For the detection of human IL-6 the coupling of the microtiter plate was performed with anti-human IL-6 coating antibody (R&D Systems,Wiesbaden, Germany). As standard recombinant human IL-6 (RD Systems) was used and the binding was detected by a secondary anti-human-IL-6 Biotin-conjugated antibody ((R&D Systems), followed by Streptavidin-HRP. As substrate TMB super sensitive (BioFX, Sur Modics) was used and the reaction was stopped by adding 1 M H_3_PO_4_ (pH 8.2). The OD was measured with an E max Precision Microplate Reader (Molecular Devices) at 450 nm and analyzed with the „Softmax Pro”software.

### Statistics

Statistical significance was analysed using Prism 4.01 GraphPad^®^ software. Means and the respective standard deviations (SD) were calculated. Significance was tested by unpaired Student‘s-*t*-test. * are P values < 0.05; ** are P values < 0.01, *** are P values < 0.001.

## Results

### IAV induces the release of BPI from human granulocytes

**Fig 1:**
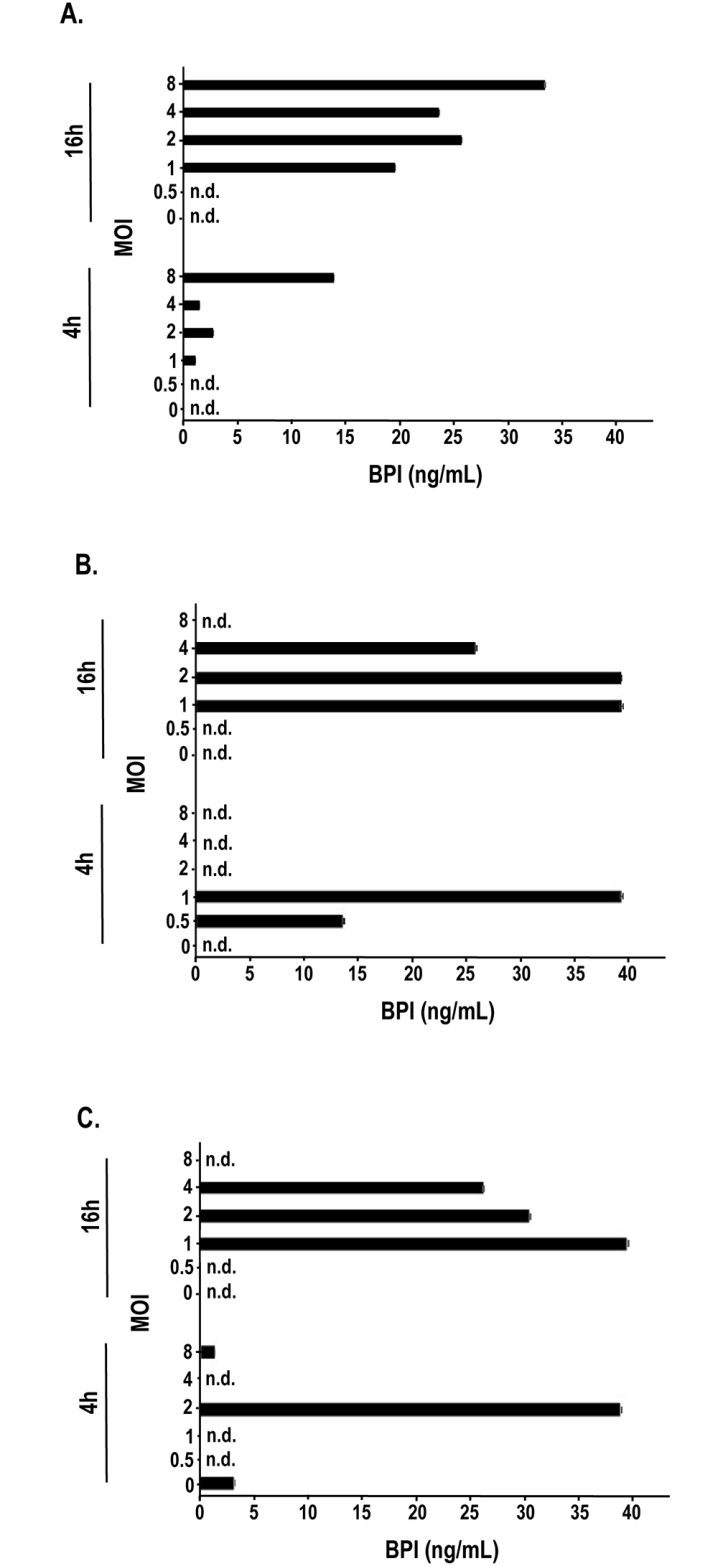
Influenza A virus induces the release of BPI from human granulocytes. After Dextran sedimentation granulocytes were isolated from human buffy coats by density gradient centrifugation. After the control of the purity of the collected neutrophils by the measurement of the surface expression of CD14 and CD66 using flow cytometry the degranulation of the granulocytes were induced in the presence of purified Influenza A virus subtypes A) H1N1, B) H3N2 and C) H5N1 (0.5, 1, 2, 4, and 8 MOI, black bar) or left unstimulated (white bar). The IAV strain rg A/Vietnam//1203/04 (H5N1) harbours only hemagglutinin as well as neuraminidase form H5N1 and the rest of the virus is of strain A/PR/8/34 origin. After 4 as well as 16 hrs of stimulation the supernatants were collected and the content of BPI in the supernatant was determined via ELISA. Three experiments with similar results were performed and one representative is shown. Statistically significant differences are given as p values (** <0.01); n = 3 ± SEM.

To determine whether the stimulation of neutrophilic granulocytes with the Influenza A virus strains H1N1 (Fig 1A), H3N2 (Fig 1B) as well as IAV strain rg A/Vietnam//1203/04 harbouring hemagglutinin and neuraminidase from H5N1 (Fig 1C) would result in the release of BPI from the azurophilic granules of these cells we isolated human neutrophilic granulocytes form buffy coats. Usually the cells had a purity of greater than >95% as judged by flow cytometry (data not shown). These cells were stimulated in the presence of 0.5–8 MOI of the indicated purified IAV strains. After 4 as well as 16 hours of stimulation the incubation of the neutrophils with virus resulted in the enhanced release of BPI (Fig 1).

### BPI inhibits the activation of PBMCs by IAV

**Fig 2:**
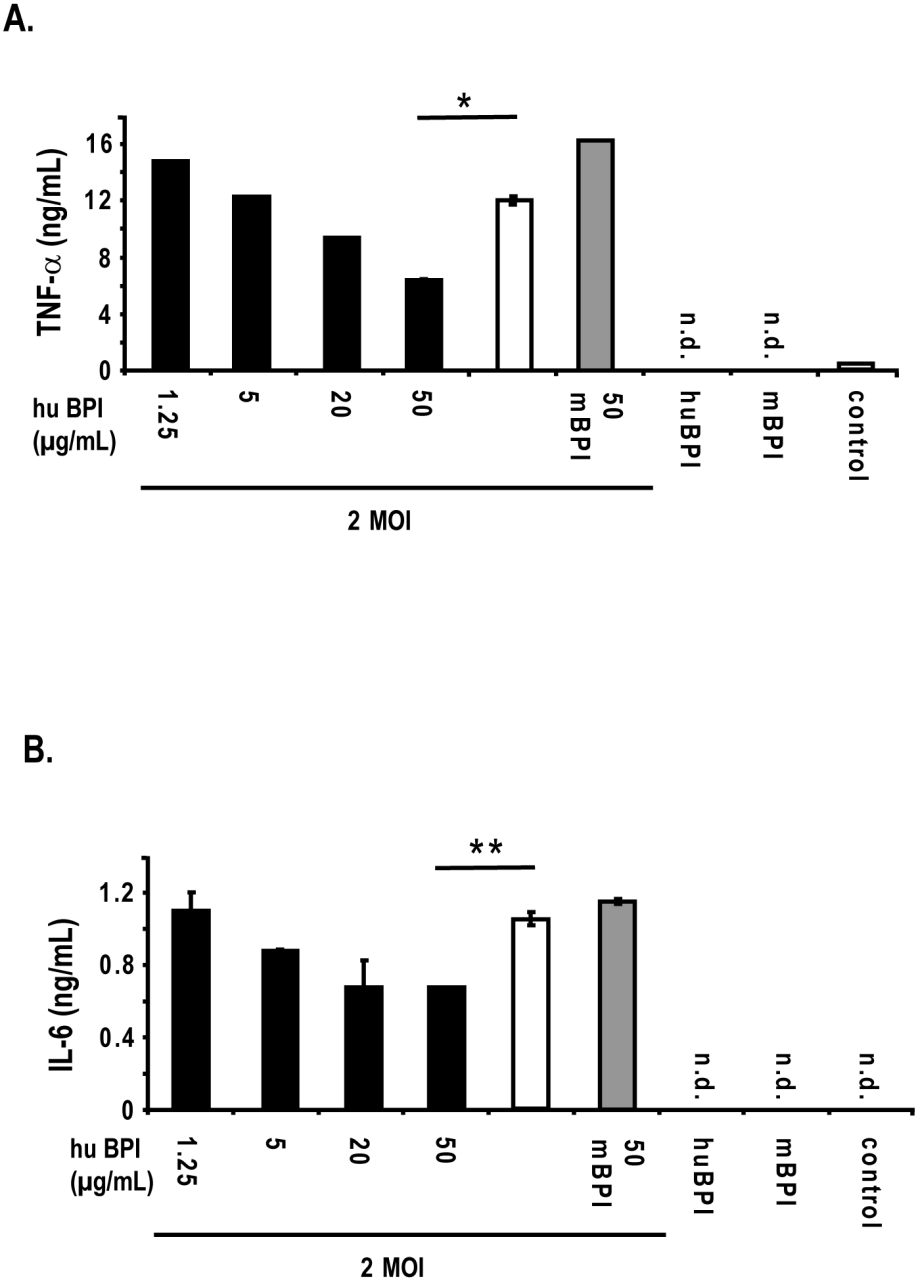
Human BPI-peptide inhibits the activation of PBMCs by Influenza A virus. Human PBMCs were isolated by dense gradient centrifugation from buffy coats. Thereafter, the cells were seeded and stimulated with purified Influenza A virus (MOI 2) (H1N1) in the presence (black bars) of increasing concentrations and absence (white bar) of human and mouse BPI-peptides (50 μg/mL, (grey bar)), and left unstimulated (control). In addition, the cells were incubated in the presence of either human BPI-peptide (50 μg/mL, huBPI) or mouse BPI-peptide (50 μg/mL, mBPI), respectively. After 20 h the supernatant was collected and the release of IFNα (A) as well as IL-6 (B) was determined by a specific ELISA. One representative experiment out of three is displayed. Statistically significant differences are given as p values (* <0.05 and ** <0.01); n = 3 ± SEM.

Since we observed an enhanced release of BPI from neutrophils after the interaction with IAV obtaining a strong response with a MOI 2 we asked whether BPI would influence the stimulation capacity of the virus. Therefore, we isolated human PBMCs from buffy coats and stimulated the cells in the presence of MOI 2 of purified Influenza A virus (H1N1). The virus were pre-incubated with increasing amounts of human BPI-peptide for 30 min, which was previously shown to harbour antibacterial activity [20], or with the respective homologous mouse BPI-peptide and added to the cells thereafter. After 20 h of infection the supernatant was collected and analysed via an IFNα as well as an IL-6 ELISA. Only the pre-incubation of IAV with human BPI-peptide did inhibit the release of IFNα (Fig 2A) as well as IL-6 (Fig 2B) from these cells in a dose dependent manner whereas the mouse BPI-peptide homolog did not show this effect. From this experiment we concluded that the human BPI-peptide does interfere with IAV infectivity

### BPI inhibits the infectivity of IAV

**Fig 3:**
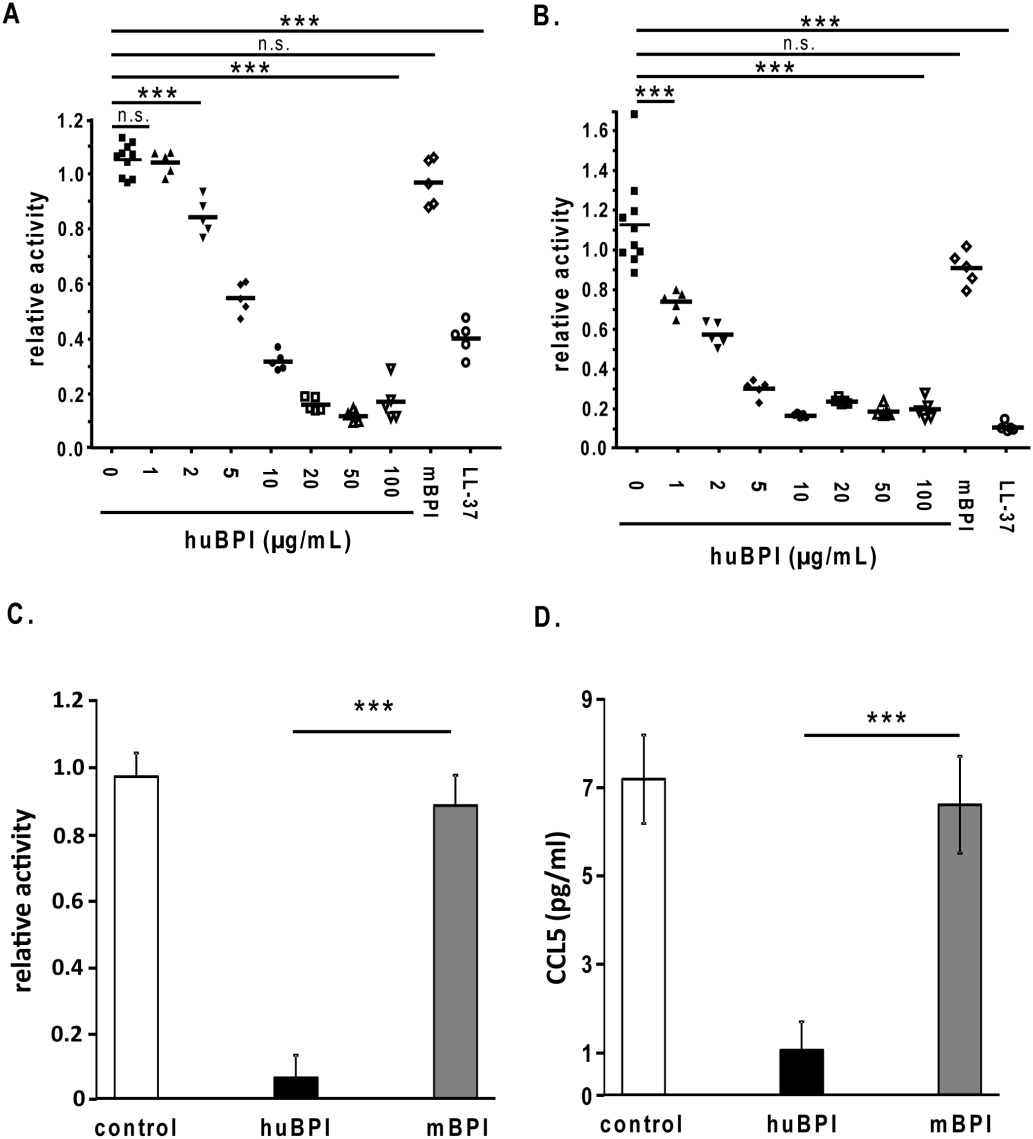
Human BPI-peptide specifically inhibits the replication of different IAV strains. Protease-deficient MDCK(H) cells were infected with 500 PFU/well of Influenza A virus strain A/PR/8/34 (H1N1) (A) and strain A/Aichi/2/68 (H3N2) (B) for 1 h. After the infection the virus containing supernatant was removed and the cells were grown for additional 13 h. Thereafter, the fixed and permeabilized cells were incubated with the mouse anti–nucleoprotein IAV monoclonal antibody. The binding of the antibody was detected by a donkey anti-mouse IgG-HRP antiserum and adding the reagent TMB Super Sensitive One Component HRP Microwell Substrate. Substrate conversion was detected by 450 nm. For the control sample is n = 8 ± SEM, all other samples n = 5 ± SEM. C) Calu-3 cells were infected with 300 PFU/well of Influenza A virus strain A/Aichi/2/68 (H3N2). Prior to the infection the virus was incubated in the presence or absences of the indicated amount of human or mouse BPI-peptide for 1 h. Thereafter, the virus solution was removed and the cells were incubated for 24 h. The virus amount in the supernatant was analysed by adding an aliquot of the supernatant to MDCK (H) cells for 1 h. After the infection the virus containing supernatant was removed and the cells were grown for additional 13 h. Thereafter, the fixed and permeabilized cells were incubated with the mouse anti–nucleoprotein IAV monoclonal antibody and detected as outlined above. (D) Furthermore, also the release of CCL5 into the supernatant of the infected Calu-3 cells was determined via a specific ELISA. One representative experiment out of 5 performed is displayed. Statistically significant differences are given as p values (** <0.01 and *** <0.001); n.s. is not significant; n = 3 ± SEM.

To get a first insight at what level of the infection cycle human BPI does interfere with IAV we chose a single round infection model. Thereby, MDCK cells harbouring a protease deficiency which will not release virus after infection were infected with 500 PFU/well IAV in the presence or absence of different peptides derived from human or mouse BPI, respectively. Therefore MDCK cells were infected with Influenza A virus strain A/PR/8/34 (H1N1) (A) and strain A/Aichi/2/68 (H3N2) (B) for 1 h. After the infection the virus containing supernatant was removed and the cells were grown for additional 13 h. We could demonstrate that human BPI-peptide specifically was able to inhibit the infection of MDCK cells since the mouse BPI-derived homologous peptide had no effect (Fig 3A). Additionally we could demonstrate that human BPI-peptide did inhibit the infectivity of different subtypes of IAV (Fig 3A for H1N1 and Fig 3B for H3N2; H5N1 both IAV strain rg A/Vietnam//1203/04 harbouring hemagglutinin and neuraminidase from H5N1, S1A Fig, as well as a clinical H5N1 isolate, S1B Fig).

After the inhibition of the IAV infection of MDCK-cells we analysed whether BPI might also inhibit the infection of the human lung epithelial cell line Calu3 with IAV. Therefore, IAV (H3N2) were incubated in the presence of either human or mouse BPI-peptide prior to infect Calu3 cells for 1 h. Thereafter, the virus solution was removed and the cells were incubated for 24 h. We were not able to detect endogenous BPI in the supernatant of IAV infected Calu3 cells (data not shown). In addition, after overnight incubation the virus titer was determined by plating serial dilutions of the supernatant on MDCK cells. As shown in Fig 3C the human BPI peptide did also inhibit the infectivity of the primary IAV target cells during infections resulting in strongly diminished virus titer. In accordance to the virus particle data human BPI-peptide did also inhibit the release of the chemokine CCL5 into the supernatant of the infected Calu3 cells (Fig 3D).

### BPI does not induce an antiviral status in the target cells

**Fig 4:**
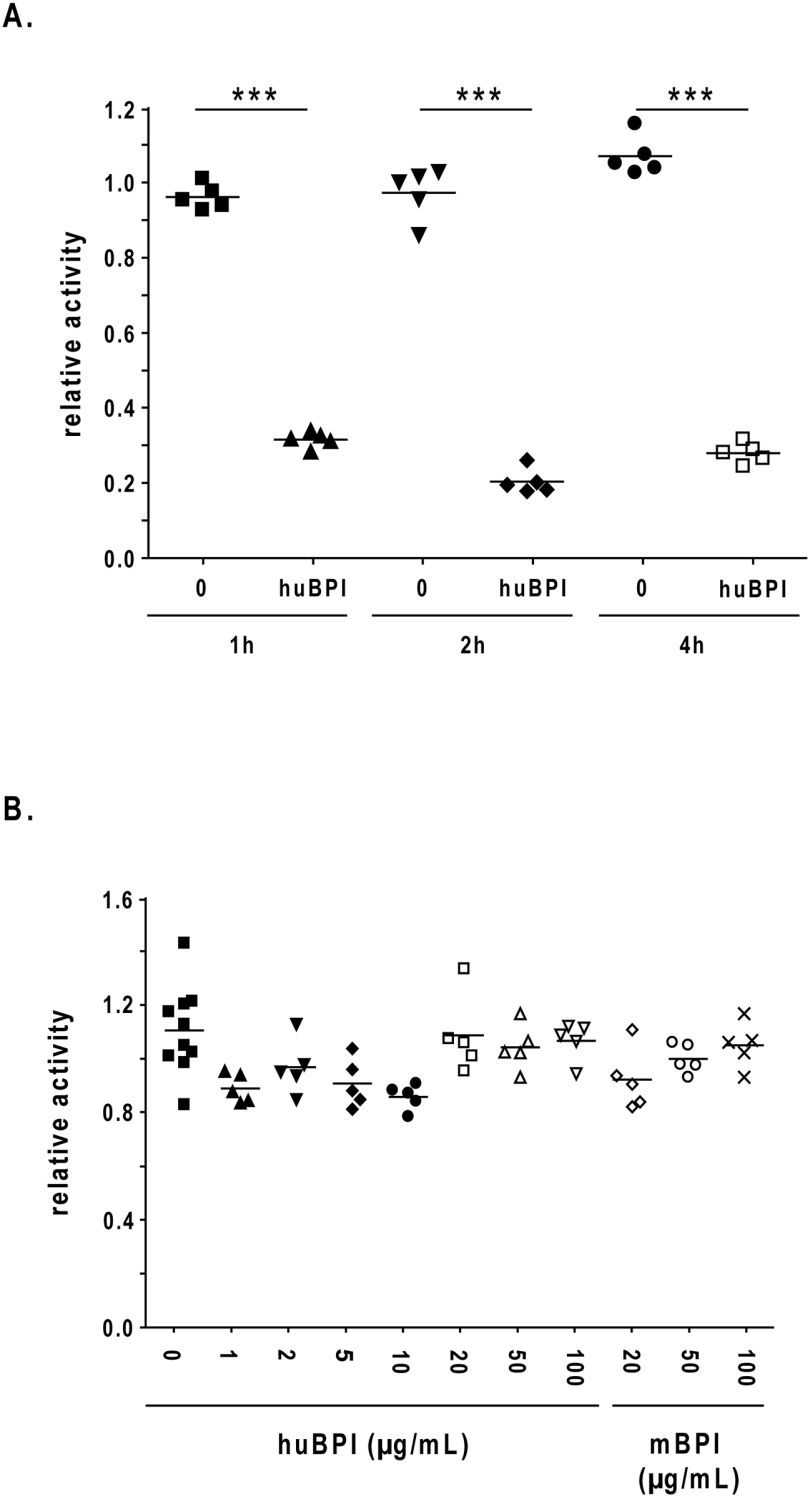
Human BPI-peptide acts not via a cell autonomous pathway. Protease-deficient MDCK(H) cells were infected with 500 of PFU/well Influenza A virus strain A/PR/8/34 (H1N1) for 1 h. Before the infection the virus was incubated in the presence or absence of the indicated amount of human BPI-peptide or control peptides for 1 h up to 4h (A). After the infection the virus containing supernatant was removed and the cells were grown for additional 13 h. Thereafter, the fixed and permeabilized cells were incubated with the mouse anti–nucleoprotein IAV monoclonal antibody. The binding of the antibody was detected by a donkey anti-mouse IgG-HRP antiserum and adding of the reagent TMB Super Sensitive One Component HRP Microwell Substrate at 450nm. (B) MDCK (H) cells were preincubated with the indicated amount of peptides for 1 h. Thereafter the supernatant of the cells was removed and the cells were infected with 500 PFU/well Influenza A virus strain A/PR/8/34 (H1N1) for 1 h. Subsequently the infection and detection of the virus was performed as described above. One representative experiment out of 3 performed is displayed. One representative experiment out of 3 performed is shown. Statistically significant differences are given as p values (*** <0.001); n = 5 ± SEM.

First we were interested whether human BPI peptide would act stronger when it was incubated for longer time periods before we added IAV (H1N1) to the target cells. We could not observe an enhanced antiviral activity of human BPI-peptide when we prolonged the virus-peptide interaction time up to 4 h (Fig 4A).

Furthermore, to get a first hint in the mechanistic action of the human BPI peptide we incubated the MDCK (H) cells with the human BPI peptide for 1 h before the peptide was removed and the cells were infected (IAV H1N1). This experimental setting did not lead to an inhibition of viral replication in the target cells (Fig 4B). We were also not able to detect the release of type I IFN from the MDCK (H) cells after peptide incubation without virus infection (data not shown). From this experiment we concluded that human BPI-peptide did not induce an antiviral status in MDCK (H) cells and therefore, the peptide might directly interact with the virus particles.

### BPI inhibits the replication of VSV

**Fig 5:**
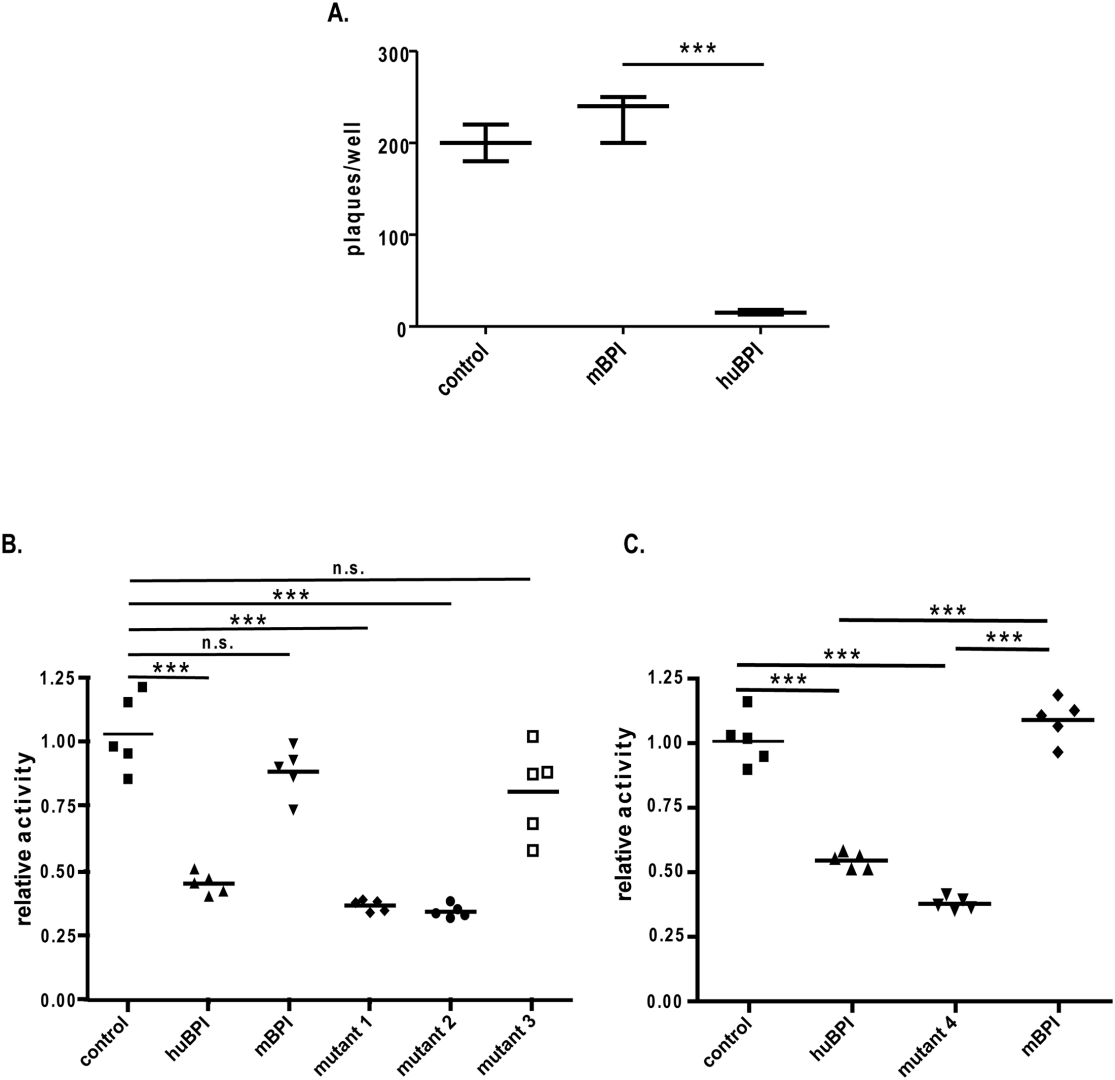
Mutant human BPI-peptide corresponding to the mouse peptide loose its activity and human BPI peptide inhibits the replication of VSV. BHK-cells were infected with 360 PFU/well of VSV-GFP virus. Prior to infection the VSV was incubated with 20 μg/mL of human BPI (huBPI) and mouse BPI-peptide (mBPI), respectively or remained untreated (control) for 1 h. After 1.5 h of the cells were overlaid with 1.25% Avicel medium and incubated for 42 h. Thereafter, the cells were fixed and stained with crystal violet for 1 h at 4°C. Finally the plaques were counted (A). MDCK(H) cells were infected with 500 PFU/well Influenza A virus strain A/PR/8/34 (H1N1) for 1 h. Before the infection the virus was incubated in the presence or absence of 20 μg/mL of either human BPI-peptide (huBPI), murine BPI-peptide (mBPI), human BPI-peptide ^N^1^D^ (mutant 1), human BPI-peptide ^K^11^M^ (mutant 2), human BPI-peptide ^N^1^D^ and ^K^11^M^ (mutant 3) or left untreated (control) for 1 h (B) or incubated in the presence or absence of 20 μg/mL of either human BPI-peptide (huBPI), murine BPI-peptide (mBPI), mouse BPI-peptide ^D^1^N^ and ^M^11^K^ (mutant 4) or left untreated (control) for 1 h (C). After the infection the virus containing supernatant was removed and the cells were grown for additional 13 h. Thereafter, the fixed and permeabilized cells were incubated with the mouse anti–nucleoprotein IAV monoclonal antibody. The binding of the antibody was detected by a donkey anti-mouse IgG-HRP antiserum and adding of the reagent TMB Super Sensitive One Component HRP Microwell Substrate. Substrate conversion was detected by 450 nm. One representative experiment out of 3 performed is shown. Statistically significant differences are given as p values (*** <0.001); n.s. is not significant; n = 5 ± SEM.

In order to analyze whether the action of the BPI-peptide was specific towards IAV we investigated the activity of the BPI-peptide against VSV infectivity. As depicted in Fig 5A we could show that the human BPI-peptide did inhibit the infectivity of VSV particles in a plaque test with BHK cells. Furthermore, we could not show that human BPI-peptide was able to inhibit the infectivity of HIV or measles virus (S2 and S3 Figs). However an inhibitory effect of human BPI against HIV could be observed (S2A Fig) but this inhibitory effect of the human BPI peptide at higher concentrations (100 and 20 μg/mL) could be attributed to the toxic effects of the peptide against the cell line used in this experiment (S2B Fig).

To get a first insight why the human BPI-peptide displayed antiviral function in comparison to the mouse homologue we compared the sequences of both peptides. Thereby we noticed that differences in the amino acid sequences between mouse and man resulted in changes in the overall charge of the peptides. Whereas the human peptide has a charge of +5 the mouse peptide has an overall charge of +3. Therefore, we inserted two mutations separately and in combination to the human peptide, which resulted in the decrease of the charge in the double mutant peptide to +3 (Table 1). As shown in the Fig 5B both mutations separately resulted in no reduction of the activity of the human peptide but the combined mutations in the human BPI-peptide led to a loss of great proportions of its activity against Influenza A virus (H3N2) (Fig 5B). Furthermore, an additional mouse peptide was generated which displayed the charge of the human wild type peptide (Table 1). The mutation of the previously inactive mouse BPI peptide in a way that it has the same charge as human BPI peptide (+5) is sufficient to convert the mouse BPI into an antiviral active peptide (Fig 5C).

### BPI modifies the structure of IAV particles

**Fig 6:**
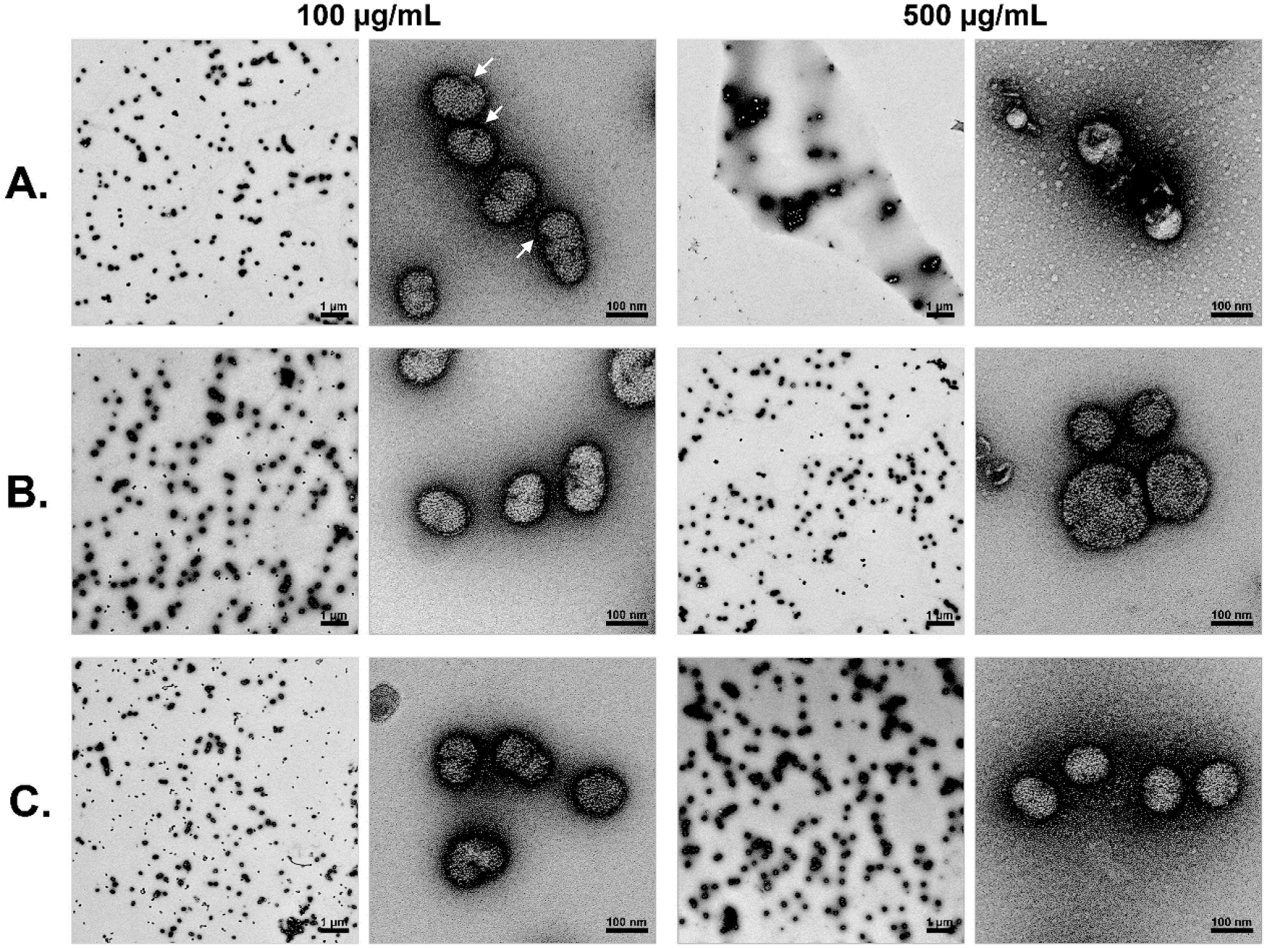
Human BPI-peptide damages the IAV particles. Virus particles were incubated either with 100 (100) and 500 μg/mL (500) of human (A) or murine BPI-peptides (B) for 1 h or left untreated (C). After the incubation the virus particles were visualized by transmission electron microscopy. Therefore, the particles were negatively stained with 2% uranylacetate and transmission electron microscopy was carried out using a JEOL TEM 2100 at 120kV. Micrographs were recorded with a fast-scan 2k x 2k CCD camera F214. One representative experiment out of 3 performed is displayed.

To get an insight of the action of the BPI peptide against IAV we analyzed the potential of human BPI peptide to inhibit the haemagglutination or hemolysis effects of Influenza A virus. We could not find a meaningful inhibition of either the haemagglutination or hemolysis activity of Influenza A virus in the presence of human BPI peptide (S4 and S5 Figs). To further analyse the potential mechanism of action of the human BPI peptide we investigated the virus particles after incubation with the peptides either with 100 (100) and 500 μg/mL (500) of human or murine BPI-peptides for 1 h or left untreated by transmission electron microscopy (Fig 6). After incubation with 500 μg/mL of the human BPI-peptide clear visible structural alterations of the virus particles could be observed as these seem to lack their virus envelope and therefore, only breakdowns of the particles were visible. At lower concentrations the human BPI-peptide induced the virus capsid to bulge which is, however, still clearly visible (arrows in Fig 6). This phenomenon was described for the action of HNP-1 an antimicrobial peptide of the α-defensin family against papillomavirus [21]. In contrast, after incubation with the mouse BPI-peptide no structural changes of the virus capsid could be observed. A similar experimental setup for VSV did not result in visible damage of the virus envelope after incubation with human BPI peptide (S6 Fig).

## Discussion

Human BPI displays a very well characterized antimicrobial activity against various gram negative bacteria. We could recently demonstrate that a 27 aa acid peptide derived from the N-terminal part of the protein is able to inhibit the growth of even multi resistant *Pseudomonas aeruginosa* very efficiently [20]. Moreover, the protein of mouse and human BPI neutralizes Lipopolysaccharide from the cell wall of gram negative bacteria with similar efficiency [22]. Most likely the killing of the bacteria results from the interference of the BPI with the cell wall of the bacteria which finally leads to an osmolaric instability of the bacterial membrane [23]. We extend the activity of human BPI to IAV as well as VS-virus. As shown by electron microscopy this happens most likely by the interaction of the BPI-peptide with the virus particle leading to direct damage of the envelope of the virus particles. This however does neither result in the inhibition of the haemagglutination activity nor in the hemolysis capacity of Influenza A virus. Since we were not able to demonstrate antiviral activity against HI-virus envelope this activity displays some sort of specificity. What exactly determines this specificity is currently unknown.

The principle activity of defensins against viruses was demonstrated before. There is an increasing body of evidence that AMPs act against a variety of viruses. For example α-defensins were shown to inhibit infections mediated by papillomavirus [21], neutralize adenovirus [24] and inhibit the fusion of HIV with PBMCs [25]. Mechanistically, it was demonstrated that the entering of HIV into its target cells was inhibited by HNP-1 at multiple steps. First the binding of the virus to its co-receptor (CCR5 und CXCR4) was blocked. Second the endocytosis of the virus into the target cell was inhibited and finally the release of the HIV -genome from the endosome into the cytoplasm was blocked. HNP-1 binds to the N-terminal part of the primary cellular receptor of HIV, namely CD4 preventing thereby the interaction of the virus with the co-receptor. At the same time HNP-1 binds to the trimeric Env of the virus [13]. Importantly the endocytosis of Influenza A virus could not be inhibited by HNP-1. On the other hand an antiviral activity against Influenza A virus mediated by HNP-1was published before [26]. The antiviral effect in this study most likely was due to the activation of the target cells with the peptide rather than a direct activity against the virus particles. We, however, did not observe any activation of the cells, e.g. granulocytes, PBMCs as well as MDCK cells by the BPI-peptides in this study. Instead we clearly demonstrate a direct effect on the virus envelope. The induction of the release of AMPs including BPI by Influenza A virus directly acting against the incoming thread might be a mechanism how the immune system restricts the infection at an early stage during the infection process and may limit pathogenicity of the pathogen.

Furthermore, the cathelicidin LL-37 was shown to inhibit the infectivity of HIV. From LL-37 it is known that it binds to the cellular formyl-peptide receptor 1 (FPRL-1) and mediate chemotaxis, immunomodulatory activity as well as angiogenic functions enabling LL-37 to activate the immune system [27]. However the anti-HIV activity was independent on the FPRL-1 [28]. Moreover LL-37 by as yet unknown reasons acts against adenovirus and Herpes simplex virus 1 and 2, respectively [29,30]. In another article the cathelicidin had direct effects on the virion structure of vaccinia virus as judged by transmission electron microscopy [31]. Finally, LL37 influenced the infectivity of Influenza A virus and the application of LL-37 to mice prevented or inhibited the infection with Influenza A virus in an infection model [12]. The molecular mechanism of how LL-37 leads to the reduced infectivity of the virus was not analyzed. Furthermore, the spectrum of the antiviral potency of LL-37 was not determined.

Another aspect of our study shows that the antiviral activity of the peptide is determined by the charge of the peptide. This corroborates our findings that the peptides directly damage the virus envelope. To do so the peptides need to directly interact and may bind to the virus envelope and therefore the charge of the peptide is an important determinant of this interaction. This is also known for the antimicrobial activity of the defensins and BPI as well. Due to the cationic nature of the AMPs these proteins are able to insert into the anionic charged bacterial membrane where they are able to make wholes into the cell membrane leading to an osmolaric instability of the bacteria [32]. Modifications of AMPs lead to changes in their tertiary structure and providing antimicrobial activity. This was shown for the β-defensin HBD-3. Reduction of the disulphate bonds in HBD-3 through the action of thioreductase unmasks its antimicrobial potential [32]. This shows that changes in the three-dimensional structure of an AMP determines the antimicrobial potential. In addition the subtle changes in the charge of the peptides resulted also in the gain or loss of activity. Further analysis especially in infection models might demonstrate whether peptides derived prom BPI displaying anti-viral activity can serve as alternatives in the future to treat infections with IAV. Furthermore the requirements in the sequence or the structure of such peptides might help to predict and design potential peptides with anti-viral effects. This might be of particular interest in the setting were treatment with conventional anti-IAV medication is not working due to mutations in the virus genome.

## Supporting Information

S1 Fig Human BPI-peptide specifically inhibits the replication of Influenza-A Vietnam (H5N1). Protease-deficient MDCK(H) cells were infected with 500 PFU/well of Influenza A-Virus strain A Vietnam (H5N1) for 1 h. A) The IAV strain rg A/Vietnam//1203/04 (H5N1) harbours only hemagglutinin as well as neuraminidase form H5N1 and the rest of the virus is of strain A/PR/8/34 origin and B) the IAV strain A/Thailand/1(Kan-1)/2004 strain (H5N1). After the infection the virus containing supernatant was removed and the cells were grown for additional 13 h in case of Vietnam (H5N1) and 8 h for Thailand (H5N1). Thereafter, the fixed and permeabilized cells were incubated with the mouse anti–nucleoprotein Influenza A monoclonal antibody. The binding of the antibody was detected by a donkey anti-mouse IgG-HRP antiserum and adding the reagent TMB Super Sensitive One Component HRP Microwell Substrate. Substrate conversion was detected by 450 nm. S ample number n = 5 ± SEM. One representative experiment out of 3 performed is displayed. Statistically significant differences are given as p values (** <0.01 and *** <0.001); n.s. is not significant.
(TIF)

S2 Fig Human BPI peptide does not inhibit the infectivity of HIV. Growth kinetics of HIV-1 in the presence of BPI. C8166 cells were infected and cultured in the presence of various concentrations of human (A) and mouse (B) BPI. Samples taken at various time-points were assayed for levels of HIV-1 Gag p24 by antigen-capture ELISA. The apparent inhibitory effect seen with the human BPI at 100μg/mL (and partially with 20 μg/mL) was the result of the high cytopathic effect of the human BPI peptide at these concentrations, even in the absence of HIV.
(TIF)

S3 Fig Human BPI peptide does not inhibit the infectivity of measles virus. 500 Pfu/well Measles virus and peptides were incubated for 1 h in a 96-well plate and thereafter Protease-deficient MDCK(H) cells were added to the virus peptide solution and incubated for additional 13 h. After that the fixed and permeabilized cells were incubated with the mouse anti–measles matrixprotein monoclonal antibody. The binding of the antibody was detected by a secondary antibody coupled to HRP (donkey anti-mouse IgG-HRP) and adding of the reagent TMB Super Sensitive One Component HRP Microwell Substrate. Substrate conversion was detected by 450 nm. Sample number n = 5 ± SEM. One representative experiment out of 3 performed is displayed.
(TIF)

S4 Fig Human BPI does not inhibit haemagglutination activity of Influenza A virus. 500 PFU/well of the IAV strains A/PR/8/34 (H1N1), strain A/Aichi/2/68 (H3N2) or strain rg A/Vietnam//1203/04 (H5N1) were incubated with 100 μg/mL of the indicated peptide for 1 h. Thereafter, 2-fold serial dilution of the peptide virus samples were made and an equal volume of 1% sheep erythrocytes were added and incubated on ice for additional 1 h. After the incubation time pictures of the plate were taken to visualize the haemagglutination properties of the virus. One representative experiment out of 3 performed is shown.
(TIF)

S5 Fig Human BPI does not inhibit the hemolysis effects of Influenza A virus. 500 PFU/well of the IAV strain A/Aichi/2/68 (H3N2) were incubated with 100 μg/mL of the indicated peptide (human BPI peptide huBPI (black triangle), mouse BPI peptide mBPI (black triangle upside down)) or left untreated (control (black square)) for 1 h. To adjust for the autofluorescence of the erythrocytes they were included in the measurement as negative control (negative (black diamond)). Thereafter, the peptide virus solution was added to 1% hematocrit of human erythrocytes and hemolysis was induced by shifting the pH to 5 with PBS/citric acid solution. The amount of hemolysis was measured in the supernatant after 20 min incubation at 37°C at an OD 405 nm A). In B) the direct effects of the peptides towards the erythrocytes was analyzed essentially as in A) but leaving the virus out. Sample number n = 9 ± SEM. One representative experiment out of 3 performed is displayed.
(TIF)

S6 Fig Human BPI-peptide does not damage VSV particles. Virus particles were incubated either with 500 μg/mL (500) of human (A) or murine BPI-peptides (B) for 1 h or left untreated (C). After the incubation the virus particles were visualized by transmission electron microscopy. Therefore, the particles were negatively stained with 2% uranylacetate and transmission electron microscopy was carried out using a JEOL TEM 2100 at 120kV. Micrographs were recorded with a fast-scan 2k x 2k CCD camera F214. One representative experiment out of 3 performed is displayed.
(TIF)

S1 File Supplemental methods. (DOCX)
