# Supplementary figures and images for: The Human Antimicrobial Protein Bactericidal/Permeability-Increasing Protein (BPI) Inhibits the Infectivity of Influenza A Virus
Source: PLoS One. 2016 Jun 6;11(6):e0156929. doi: 10.1371/journal.pone.0156929 (PMC4894568; doi:10.1371/journal.pone.0156929)

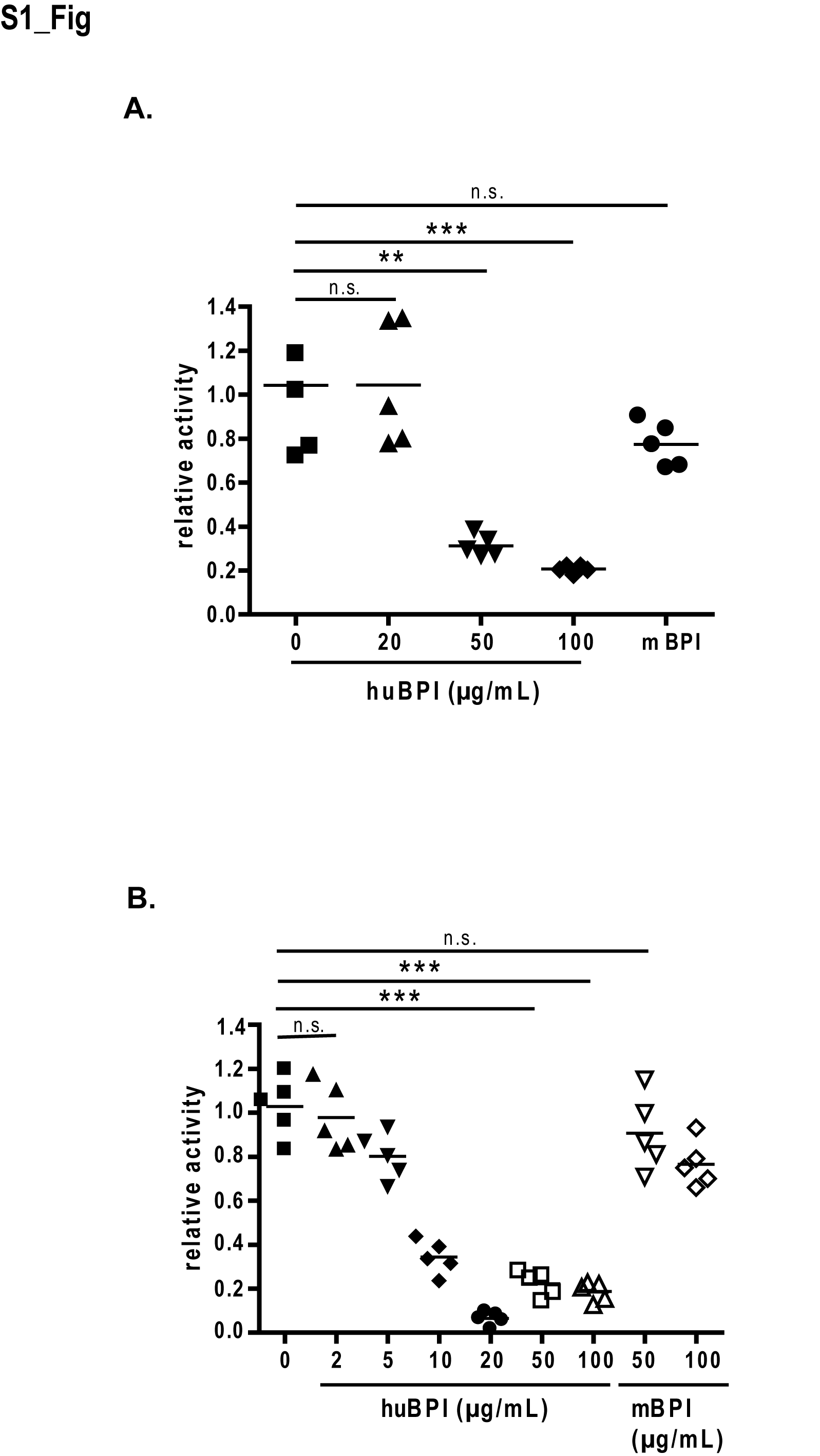

Supplement: S1 Fig — Protease-deficient MDCK(H) cells were infected with 500 PFU/well of Influenza A-Virus strain A Vietnam (H5N1) for 1 h. A) The IAV strain rg A/Vietnam//1203/04 (H5N1) harbours only hemagglutinin as well as neuraminidase form H5N1 and the rest of the virus is of strain A/PR/8/34 origin and B) the IAV strain A/Thailand/1(Kan-1)/2004 strain (H5N1). After the infection the virus containing supernatant was removed and the cells were grown for additional 13 h in case of Vietnam (H5N1) and 8 h for Thailand (H5N1). Thereafter, the fixed and permeabilized cells were incubated with the mouse anti–nucleoprotein Influenza A monoclonal antibody. The binding of the antibody was detected by a donkey anti-mouse IgG-HRP antiserum and adding the reagent TMB Super Sensitive One Component HRP Microwell Substrate. Substrate conversion was detected by 450 nm. S ample number n = 5 ± SEM. One representative experiment out of 3 performed is displayed. Statistically significant differences are given as p values (** <0.01 and *** <0.001); n.s. is not significant. (TIF) [file pone.0156929.s001.tif]

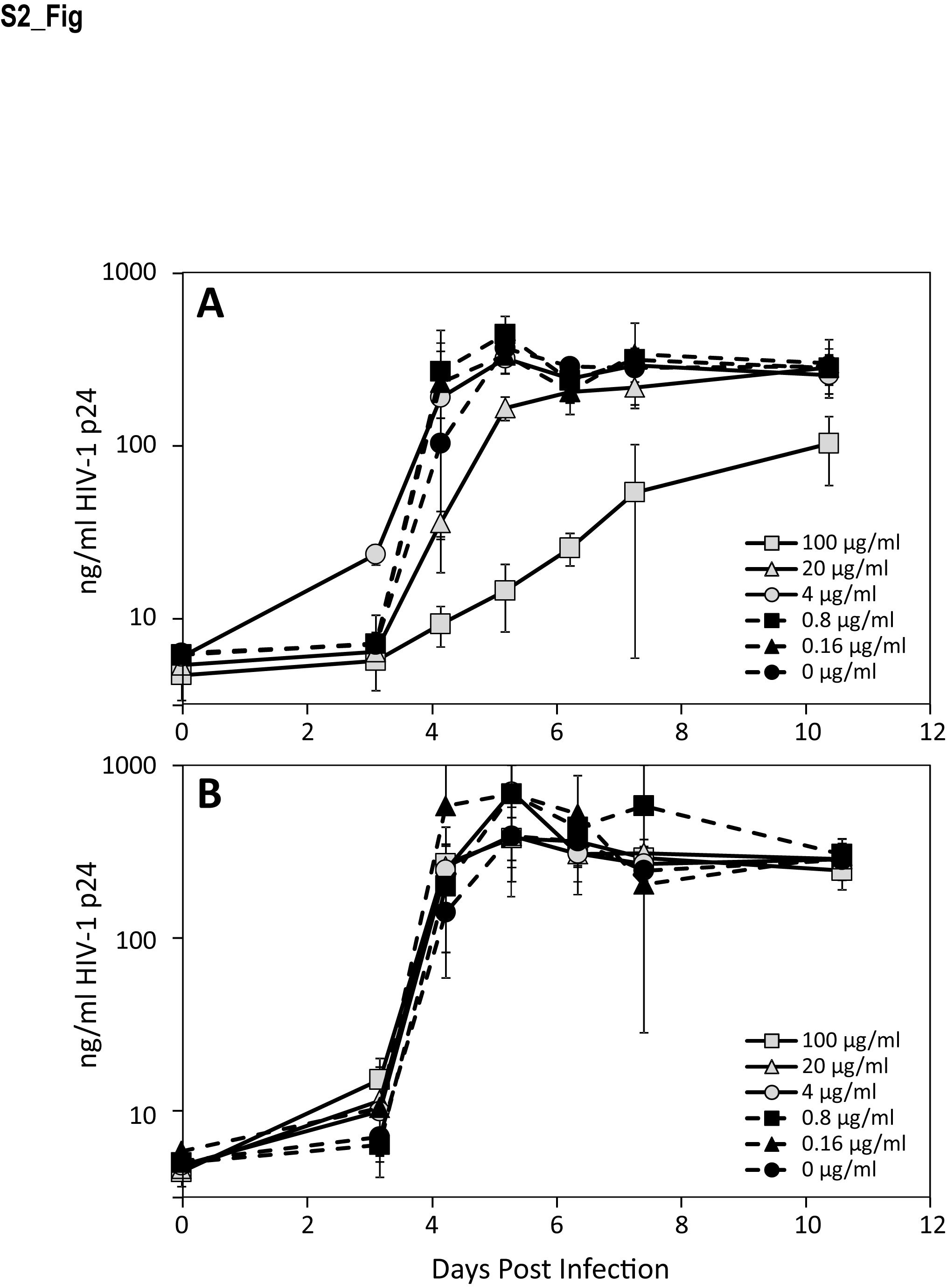

Supplement: S2 Fig — Growth kinetics of HIV-1 in the presence of BPI. C8166 cells were infected and cultured in the presence of various concentrations of human (A) and mouse (B) BPI. Samples taken at various time-points were assayed for levels of HIV-1 Gag p24 by antigen-capture ELISA. The apparent inhibitory effect seen with the human BPI at 100μg/mL (and partially with 20 μg/mL) was the result of the high cytopathic effect of the human BPI peptide at these concentrations, even in the absence of HIV. (TIF) [file pone.0156929.s002.tif]

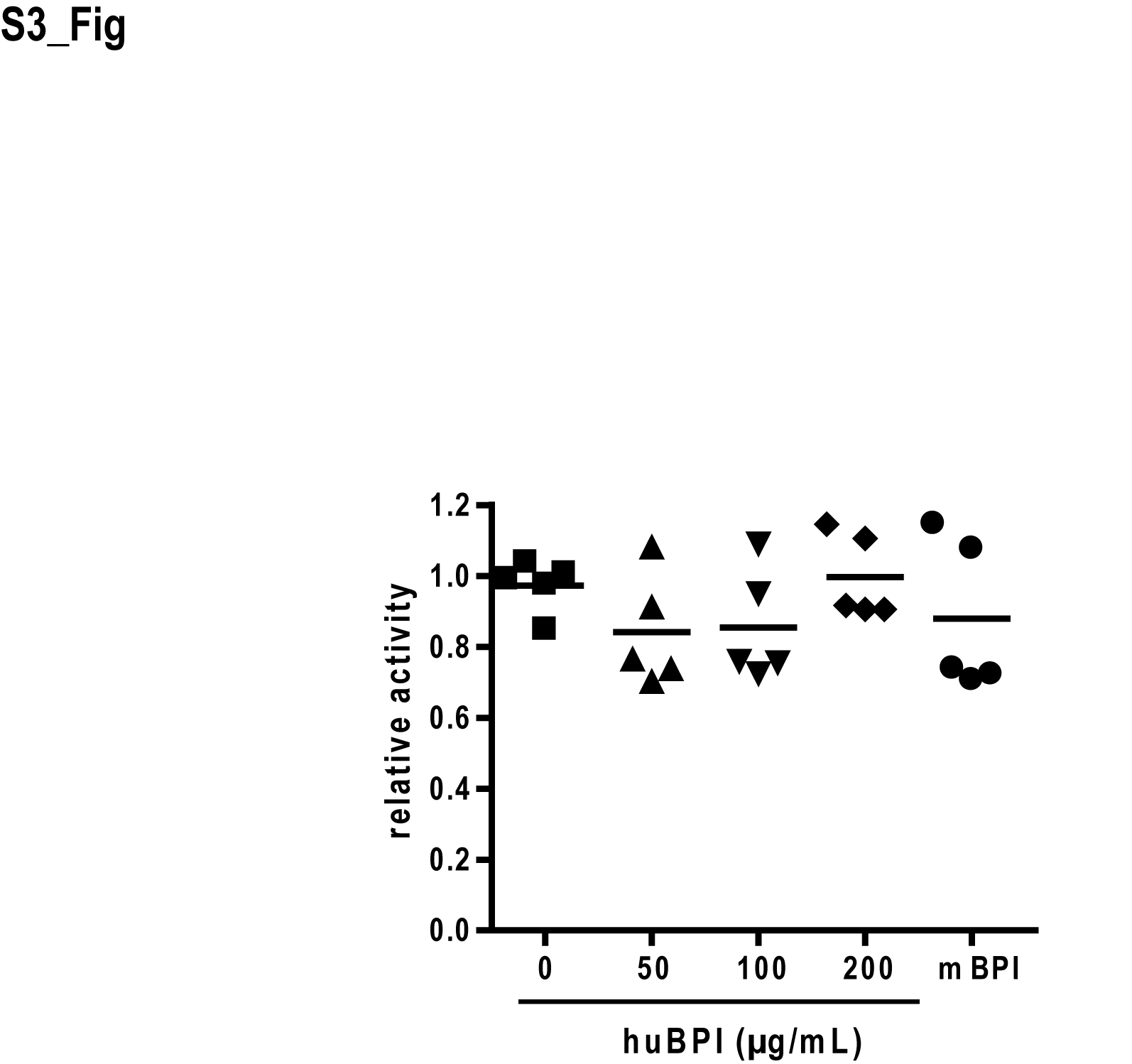

Supplement: S3 Fig — 500 Pfu/well Measles virus and peptides were incubated for 1 h in a 96-well plate and thereafter Protease-deficient MDCK(H) cells were added to the virus peptide solution and incubated for additional 13 h. After that the fixed and permeabilized cells were incubated with the mouse anti–measles matrixprotein monoclonal antibody. The binding of the antibody was detected by a secondary antibody coupled to HRP (donkey anti-mouse IgG-HRP) and adding of the reagent TMB Super Sensitive One Component HRP Microwell Substrate. Substrate conversion was detected by 450 nm. Sample number n = 5 ± SEM. One representative experiment out of 3 performed is displayed. (TIF) [file pone.0156929.s003.tif]

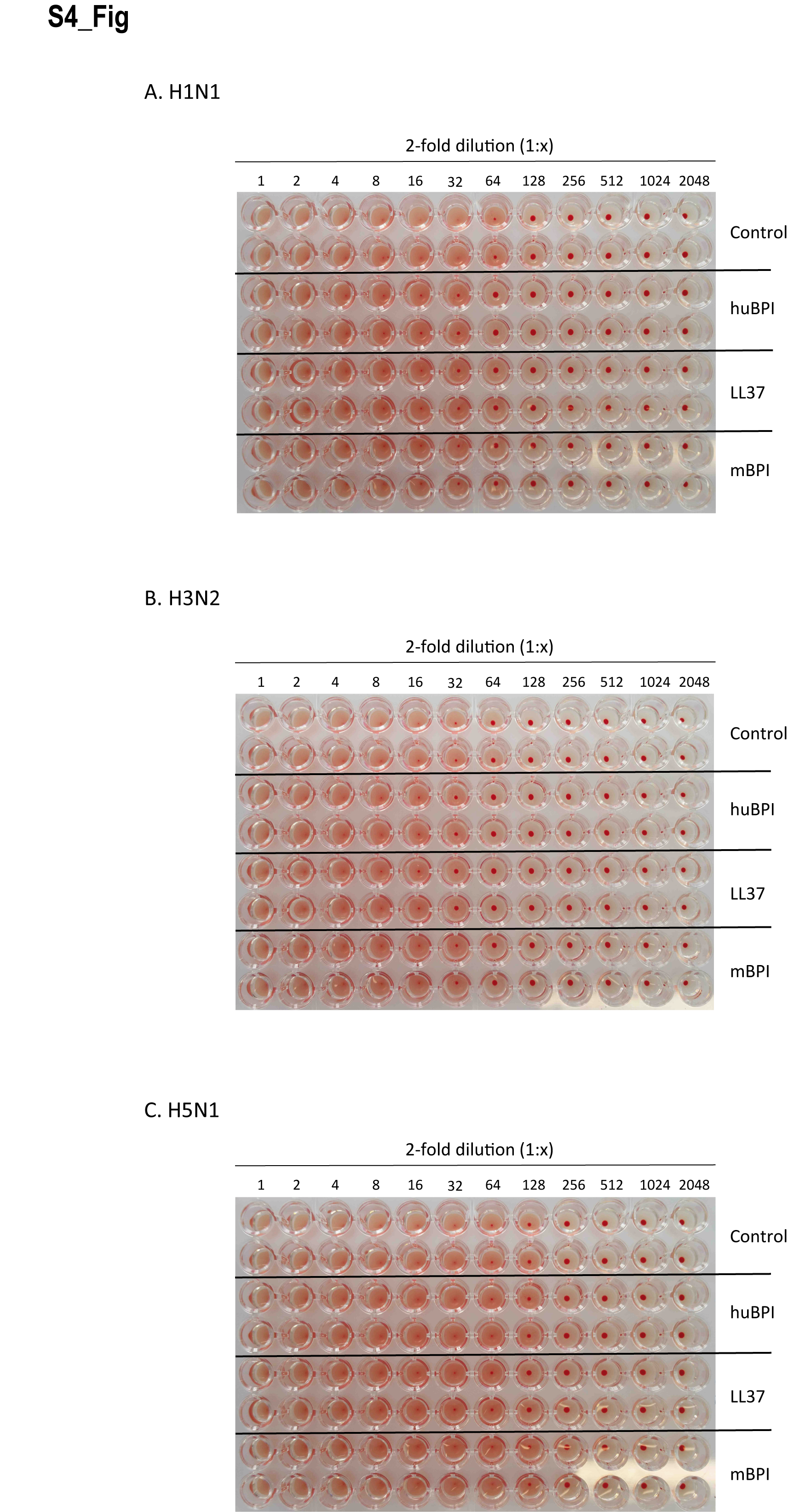

Supplement: S4 Fig — 500 PFU/well of the IAV strains A/PR/8/34 (H1N1), strain A/Aichi/2/68 (H3N2) or strain rg A/Vietnam//1203/04 (H5N1) were incubated with 100 μg/mL of the indicated peptide for 1 h. Thereafter, 2-fold serial dilution of the peptide virus samples were made and an equal volume of 1% sheep erythrocytes were added and incubated on ice for additional 1 h. After the incubation time pictures of the plate were taken to visualize the haemagglutination properties of the virus. One representative experiment out of 3 performed is shown. (TIF) [file pone.0156929.s004.tif]

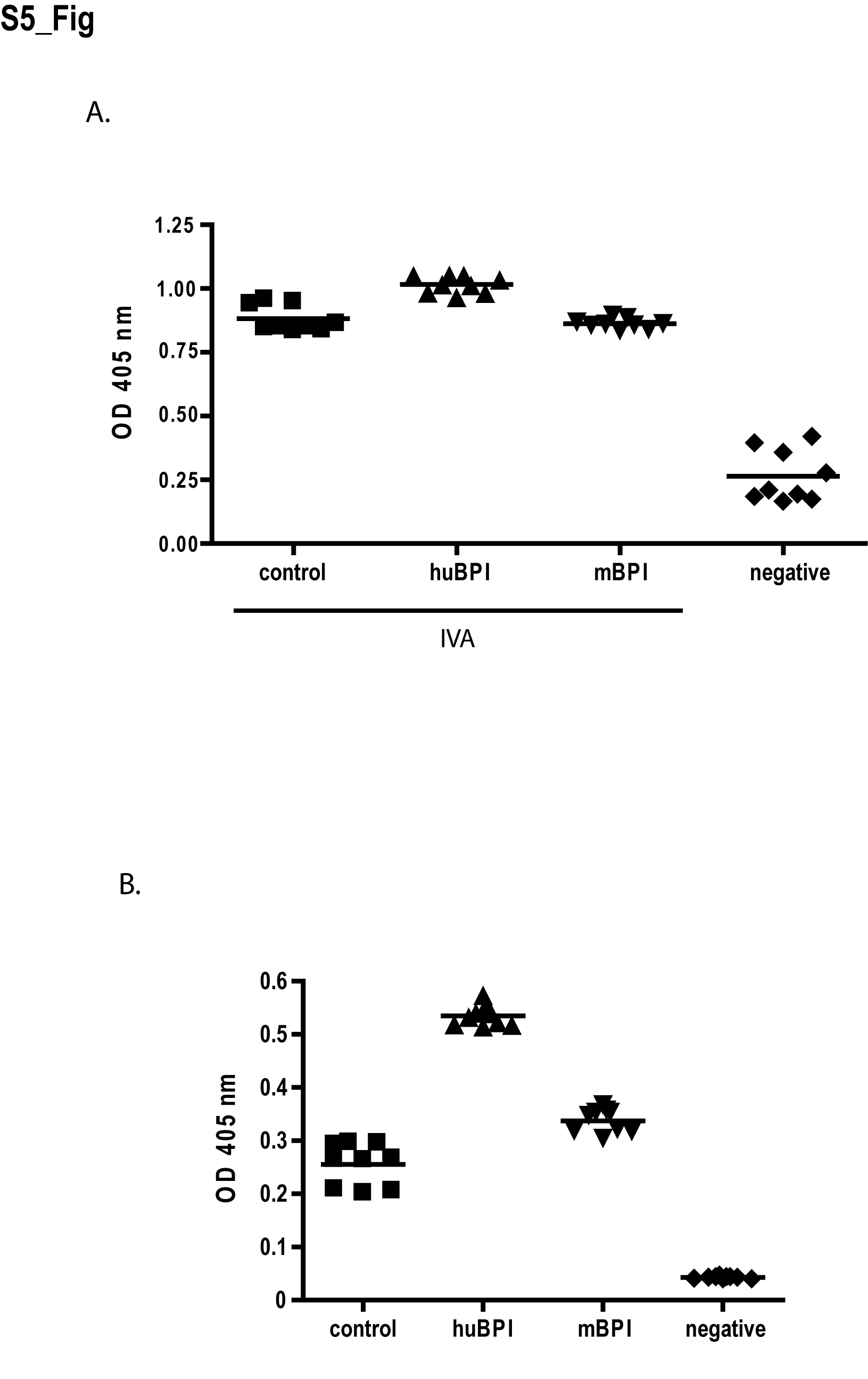

Supplement: S5 Fig — 500 PFU/well of the IAV strain A/Aichi/2/68 (H3N2) were incubated with 100 μg/mL of the indicated peptide (human BPI peptide huBPI (black triangle), mouse BPI peptide mBPI (black triangle upside down)) or left untreated (control (black square)) for 1 h. To adjust for the autofluorescence of the erythrocytes they were included in the measurement as negative control (negative (black diamond)). Thereafter, the peptide virus solution was added to 1% hematocrit of human erythrocytes and hemolysis was induced by shifting the pH to 5 with PBS/citric acid solution. The amount of hemolysis was measured in the supernatant after 20 min incubation at 37°C at an OD 405 nm A). In B) the direct effects of the peptides towards the erythrocytes was analyzed essentially as in A) but leaving the virus out. Sample number n = 9 ± SEM. One representative experiment out of 3 performed is displayed. (TIF) [file pone.0156929.s005.tif]

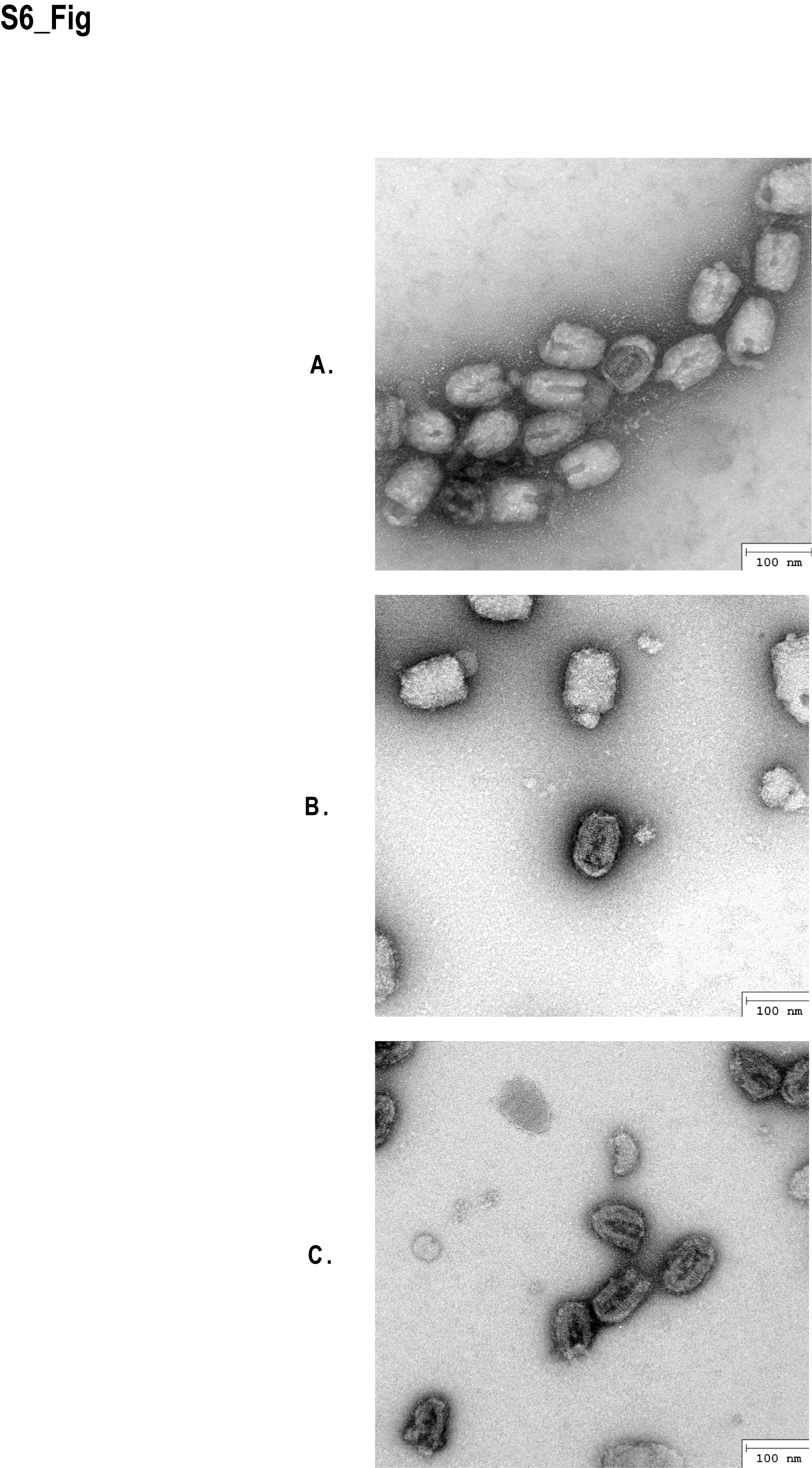

Supplement: S6 Fig — Virus particles were incubated either with 500 μg/mL (500) of human (A) or murine BPI-peptides (B) for 1 h or left untreated (C). After the incubation the virus particles were visualized by transmission electron microscopy. Therefore, the particles were negatively stained with 2% uranylacetate and transmission electron microscopy was carried out using a JEOL TEM 2100 at 120kV. Micrographs were recorded with a fast-scan 2k x 2k CCD camera F214. One representative experiment out of 3 performed is displayed. (TIF) [file pone.0156929.s006.tif]
