# Supplementary material for: The Human Antimicrobial Protein Bactericidal/Permeability-Increasing Protein (BPI) Inhibits the Infectivity of Influenza A Virus
Source: PLoS One. 2016 Jun 6;11(6):e0156929. doi: 10.1371/journal.pone.0156929 (PMC4894568; doi:10.1371/journal.pone.0156929)
Supplement: S1 File — (DOCX) [file pone.0156929.s007.docx]

**S1 File. Supplemental Method**

**HIV infection assay**

The peptides diluted in serum-free RPMI 1640 medium to give a range of final concentrations ranging from 0.16 µg/mL to 100 µg/mL were incubated in U-well microtiter plates with 500 TCID_50_ of HIV-1mn for 1 hour at 37°C before the addition of 4000 cells/well of C8166 cells (also in serum-free medium). After a further two hours incubation, wells were fed with an equal volume of RPMI 20% FCS to give a final concentration of 10% FCS.

On days 0, 3, 4, 5, 6, 7 and 10, the cells in each well were resuspended and a 10 µL sample removed and stored at -20°C for later measurement of HIV-1 Gag p24 concentration. The cell cultures in each well were also examined under the microscope for cytopathic effect and photographed at each time point.

**Measles virus infection**

Measles virus strain Edmonston was obtained from the cell culture supernatant of infected Vero cells and highly purified by ultra centrifugation. The virus titer was determined by infection of protease-deficient MDCK(H) cells. Only freshly plated MDCK(H) cells can be infected by measles virus. 500 PFU/ well of Measles virus and peptides were incubated for 1 h in a 96-well plate and thereafter the cells were added to the virus peptide solution. After the infection the cells were incubated for additional 13 h. Thereafter, the multiplied virus could be visualized inside the infected cells by the detection of the viral matrixprotein. Prior to the staining the cells were fixed with 4% paraformaldehyde and permeabilized with 0.3 % of Triton-X-100. After that the fixed and permeabilized cells were incubated with the mouse anti–measles matrixprotein monoclonal antibody (MAB8910, Millipore). The binding of the antibody was detected by a secondary antibody coupled to HRP (donkey anti-mouse IgG-HRP, dianova) and adding of the reagent TMB Super Sensitive One Component HRP Microwell Substrate (BioFX). Colour development was stopped by adding 1 M H_3_PO_4_ and detected with an E max Precision Microplate Reader (Molecular Devices) at 450 nm and analyzed with the „Softmax Pro“ software.

**Haemagglutinationstest**

The peptides (100 µg/mL) or as a control PBS pH 7.4 were incubated with either Influenza A-Virus strain A/PR/8/34 (H1N1), strain A/Aichi/2/68 (H3N2) or strain rg A/Vietnam//1203/04 (H5N1) for 1 h. Thereafter a serial dilution (1:2) of the peptide and the virus was conducted in a 96—well plate to obtain a total of 12 dilution steps to 1:2048 in 50 µL. Finally 50 µL of 1% sheep erythrocytes were added and incubated on ice for 1 h. Pictures were taken to visualize the results.

**Haemolysis assay**

The peptides (100 µg/mL) or as a control PBS pH 7.4 were incubated with Influenza A-Virus strain A/Aichi/2/68 (H3N2) for 1 h. Thereafter the virus was incubated with human erythrocytes (1 % haematocrit) for 10 min on ice. The cells should agglutinate. 0.5 mL aliquots were transferred into 1.5 mL Eppendorf tubes and pelleted by centrifugation (1000 rpm for 1min). The supernatant was abolished and the cells were incubated with either PBS with pH7 or pH5. The pH was adjusted with 0.15 M citric acid. Finally the haemolysis was determined by measuring the OD 405 nm of the supernatant. 100 % haemolysis was achieved by incubating the erythrocytes with 0.5 % Brij 36T. As negative control served a sample of erythrocytes with not virus. In order to determine the effect of the peptides directly towards the erythrocyte the experiment was conducted in absence of virus as well.
